# Journal Club presentation in research orientation at Bahria University Medical & Dental College

**DOI:** 10.12669/pjms.311.6314

**Published:** 2015

**Authors:** Rehana Rehman, Rabiya Rehan, Ambreen Usmani

**Affiliations:** 1 Dr. Rehana Rehman, Assistant Professor of Physiology, Bahria University Medical and Dental College, Karachi, Pakistan.; 2 Dr. Rabiya Rehan, Senior Lecturer, Department of Physiology, Bahria University Medical and Dental College, Karachi, Pakistan.; 3 Dr. Ambreen Usmani, Associate Professor of Anatomy, Bahria University Medical and Dental College, Karachi, Pakistan.

**Keywords:** Structured meeting, Journal club, Faculty development

## Abstract

**Objective:**

To determine faculty perception on journal club (JC) presentation at Bahria University Medical and Dental College (BUMDC), Karachi. Pakistan.

**Methods:**

It was a cross sectional study conducted from January 2009 to December 2012 to acquire faculty member’s feedback on JC presentations in structured meeting at BUMDC. Feedback was acquired by a self-reported questionnaire on a 3-pt Likert scale with a score of 1= disagree, 2= neutral, 3 = agree. Respondents were divided into Group I; senior faculty (professors, associates and assistants) and Group II of junior faculty (lecturers). Chi square test was applied to compare categorical variables; results considered significant with p value< 0.05.

**Result:**

A total of 75JC presentations were made in study period. In Group I, response was acquired by 5 Professors, 3 Associate Professors and 7 Assistant Professors whereas 34 lecturers comprised of Group II. Both groups responded to usefulness of JC equally without any significant difference. JC encouraged literature search in 35(72%), enabled 38(78%) to recall their knowledge and 34(70%) to understand study objectives. The participants 34(70%) were able to comprehend research methodology, 19(38%) understood biostatistics and 29(59%) evaluated the paper critically. The exercise motivated 36(74%) and 30(62%) participants were able to design their research projects.

**Conclusions:**

Orientation of research at BUMDC was made possible by JC discussions which encouraged literature review from reputable journals, understanding of research methodology and critical appraisals that facilitated formulation of research plans.

## INTRODUCTION

Medical education has elaborated and developed to become a discipline in its own right, place, control and resolution. It is committed to accept educational paradigm in terms of evidence- based medicine with excellence through different workshops and structured meetings.^1^^,^^2^ At the same time, there is now increasing pressure for the professionalization of teaching practice, research and its documentation, publications, oral and poster presentation in all fields of life.

A journal club (JC) is defined as “group of individuals who meet regularly to discuss critically the clinical applicability of articles in the current medical journal”.^3^ The first record of a JC was found in 1875 by Sir William Osler at McGill University for the purchase and distribution of periodicals to which he could not afford to subscribe as an individual^1^.More recently, in postgraduate medical education, JC have become a roundtable forum to teach, facilitate, enhance and enrich understanding of medical literature. The method has emerged to promote practice of evidence-based medicine (EBM) through presentation, and discussion in the form of questions and answers.^4^^,^^5^

The JC has proved to be a multipurpose academic program with a well-defined format to help in the development of many essential skills^6^.Critical appraisals JC models are extensively used to achieve defined learning objectives. Evidence based JC is used by practitioners to critic the evidence to bring a change in practice. The practice of JC however can be adjusted and formatted according to the defined target of the organizing authority and the learning objectives of the participants.^6^ This activity can have flexibility, adaptability, instructive multiplicity and contemporary pertinence.^7^

In order to establish practice of JC, a format is required to make it educational, interesting and thought provoking for all the participants. The medical educationists at Bahria University Medical and Dental College (BUMDC) accommodated the practice of presentation of JC in structured meetings since the time of inception.^1^ The strength and weakness of all innovations in medical education can be acquired from feed backs obtained from stakeholders.^8^^,^^9^ The respondents; junior and senior faculty members differed in qualification and experience and we wanted to have perception of both groups. The rationale was not only to assess usefulness of JC but to use this information to develop, modify, and improve the activity with respect to the deficiencies documented by the respondents.^10^^-^^12^ In this regards, objective of the study was to receive feedback of the faculty on the usefulness of presentations in JC.

## METHODS

It was a cross sectional study conducted from January 2009 to December 2012 to acquire faculty member’s feedback on JC presentation in structured meeting at BUMDC. The JC meetings were held every second week with the following protocol; article should be recent, from a reputable journal selected on the basis of clinical relevance, scientific merit and implantation potential. All faculty members were included and the presentation schedule was twice a year in the initial years and once in a year from 2011 to 2012. The JC presentation list was sent to all departments ahead of time. The article to be presented was uploaded on university web site and presentation was uploaded a week before. They were recommended to show and approve their power point presentation from respective Head of departments. All the Professors were excluded from presentation of JC; however they were included in the feedback response for evaluation of usefulness of JC presentation. This was made possible by a self-reported questionnaire, responses acquired on a 3-pt Likert scale with a score of 1= disagree, 2= neutral, 3= agree. The respondents were divided into two groups. Group I comprised of senior faculty which included Professors, Associate and Assistant Professors whereas Group II comprised of lecturers only. Responses of both groups were analyzed by SPSS version 15; compared by application of chi-square test, results were labeled significant with p value <0.05.

## RESULTS

The response was acquired at 98% response rate from 15 members of Group I; 5 Professors, 3 Associate Professors, 7 Assistant Professors whereas 34 lecturers comprised Group II. Total number of 75 JC presentations; 5, 9, 24 and 27 in the study period from 2009 till 2012 respectively. The responses acquired by all faculty members (Table-I) emphasize role of JC in literature search 35(71%). Presentations helped 34(69%) participants to comprehend data of discussed article, and evaluate it critically 29(59%). Biostatistics was understood by 19(39%), research motivation was developed in 36(73%) and 30(61%) participants were able to design their research projects. Both groups; I (senior) and II (junior) responded to usefulness of JC equally without any significant difference. (Table-II)

## DISCUSSION

Medical education provides road map for medical literature however, there is no standard practice and process of conducting and administrating an effective JC presentation in majority of institutions.^13^ In BUMDC educational meetings were used as a platform for a number of agendas like presentation of JC, case discussion for problem based learning and debate on curriculum development. Usmani et al. observed that only Group I of senior faculty members agreed to usefulness of these meetings in terms of social interaction, provision of learning opportunities, self-awareness, promotion of presentation skills, personal productivity and acceptance to listen to criticism.^1^ The presentation of JC in these meetings however was approved by both senior as well as junior participants in terms of its usefulness for research orientation, motivation and useful participation.

Studies have documented that JC presentations improve reading habits, review of literature and understanding of articles with sufficient level of challenge for self-directed learning.^14^^,^^15^ Our study has showed that JC encouraged literature search in 72% participants whereas in another study Douglas et al. observed 62% participants got information about current literature in Orthopedic Residency Programmes.^2^ The JC presentations in our survey enabled more than half of the participants to acquire knowledge of presented research paper with the help of debate, discussion and question answer sessions. JC can thus develop the habit of staying updated on the significant literature published every year in medical science.^16^

EBM is the process of systematically reviewing, appraising and using clinical research findings to aid the delivery of optimum clinical care to patients. JC activity is well established to fill the gap between research and practice, hence facilitating improved practice of EBM which directly results in better patient care.^13^^,^^17^^-^^19^ Critical appraisal is the most important component of EBM to evaluate validity and applicability of a research with respect to a problem or situation.^2^^,^^18^^,^^20^ In our study majority (59%) of faculty was able to evaluate the presented topics critically. The study is comparable to kitchens and Pfeiffer which tested effectiveness of JC in teaching critical appraisal skills, and orientation of clinical epidemiology in internal medical residents.^21^ The well-defined critical appraisal JC gained popularity and helped in the preparation of Part II MRCP psychiatry.^20^

The growth and improvement of a medical institute requires competent and supportive institutional leadership, appropriate resource allotment and advancement in the field of medical education.^2^^,^^12^ The use of JC for faculty training programs has been adopted by a number of institutions to inculcate responsiveness and skills among the members in their respective fields of interest.^6^^,^^19^^,^^22^ The professionalism and positive attitude motivates participants to overcome the barriers of research, take part in discussion, design research projects and progress as a researcher.^4^^,^^14^ This motivation encouraged faculty members to design their research plans which eventually are required for continuing professional education of participants.

**Table-I:** Responses on usefulness of journal club presentation by all faculty members

**Variables**	**Yes n(%)**
Encouraged literature search	35 (71)
Helped in recall and recognition of literature	38 (78)
Helped in understanding of discussed article	34 (69)
Helped in critical analysis	29 (59)
Helped in understanding of biostatistics	19 (39)
Acquired knowledge of recent researches	27 (55)
Helped to consult journals of repute	35 (71)
Motivated research	36 (73)
Helped in designing of research plans	30 (61)

N=49, Values are numbers percentages in parenthesis.

**Table-II:** Comparison of usefulness of Journal clubs among senior and junior faculty members

**Objectives**	**Responses**	**DA**	**N**	**A**	**P value**
Encouraged literature search	Senior	3 (20)	1(7)	11 (73)	0.23
	Junior	3 (9)	7(21)	24(71	
Research Papers presentation helped in recall and recognition of knowledge	Senior	2(13)	2(13)	11(73)	0.77
	Junior	2(6)	5(15)	27(79)	
Helped in comprehending material and data of discussed article	Senior	1 (7)	2(13)	12(80)	0.12
	Junior	1(3 )	11(32)	22(65)	
Enhanced critical appraisals	Senior	2 (13)	2(13)	11(73)	0.51
	Junior	4(12)	12(35)	18(53)	
Improved understanding of biostatistics	Senior	6(40)	4(27)	5(33)	0.90
	Junior	9(26)	11(32)	14(41)	
Encouraged knowledge in recent researches and development	Senior	3(20)	4 (27)	8(53)	0.24
	Junior	8(24)	7(21)	19(56)	
Encouraged one to consult journals of repute	Senior	1(7)	3(20)	11(73)	0.31
	Junior	4(12)	6(18)	24(71)	
Motivated research	Senior	2 (13)	2(13)	11(73)	0.11
	Junior	2 (6)	7(21)	25(74)	
Presentation helped to design/construct research plans	Senior	3(20)	4(27)	8(53)	0.67
	Junior	6(18)	6(18)	22(65)	

n=49, Values are numbers and percentages are in ()

A: agree

N: neutral

DA; disagree

Responses compared by chi square test

The study is limited in terms of validity of questionnaire and arbitrary division of study groups. Likewise, study has not pointed any confounding factors which could have assisted in the development of research plans. This however is the first study done on usefulness of JC with responses taken from a wide spectrum of participants which recommends continuation of this activity in medical institutes.

## CONCLUSIONS

JC presentations in structured meetings at BUMDC helped the faculty members to present research articles on a plat form which was appreciated by senior and junior colleagues. Research orientation was provided by literature review from reputable journals with understanding of research methodology, critical appraisals and designing of research plans. The orientation of research disseminated the knowledge with development of presentation, critical appraisal and communication skills among the participants.

## Authors’ Contribution:

***Rehana Rehman: ***Took part in study design, acquisition of data, interpretation of data, drafting the article and revising it critically for important intellectual content.

***Rabiya Rehan: ***Took part in compilation of write up and formulation of tables.

***Ambreen Usmani: ***Took part in compilation of write up, drafting the article and revising it critically for important intellectual content.

## Notes

***Recommendations: ***Formal training through workshops and continuing medical education should be arranged so as to conduct effective JC meetings.. The practice of JC presentations should be reinforced in all organizations focused on well-defined objectives, critical appraisal checklist, achievable targets and needs of participants with an attempt to maintain interest of participants.
